# Supplementary material for: Availability of Metribuzin-Loaded Polymeric Nanoparticles in Different Soil Systems: An Important Study on the Development of Safe Nanoherbicides
Source: Plants (Basel). 2022 Dec 4;11(23):3366. doi: 10.3390/plants11233366 (PMC9737720; doi:10.3390/plants11233366)
Supplement: Supplementary file 1 [file plants-11-03366-s001.zip › plants-2053240-supplementary.pdf]

plants

MDPI

Article

Metribuzin polymeric nanoparticle availability and safety application in different soil systems

Vanessa Takeshita[1]* Gustavo Vinicios Munhoz-Garcia[1], Camila Werk Pinácio[1], Brian Cintra Cardoso[1], Daniel Nalin[1], Valdemar Luiz Tornisielo[1] and Leonardo Fernandes Fraceto[2]

[1] Center of Nuclear Energy in Agriculture, University of São Paulo, Av. Centenário 303, 13400-970, Piracicaba,SP, Brazil 1; vanessatakeshita@usp.br, gvmgarcia@usp.br, camilawerk043@usp.br, brianccardoso@usp.br, danielnalin@usp.br, vltornis@cena.usp.br

[2] Institute of Science and Technology, São Paulo State University (UNESP), Av. Três de Março 511, 18087-180, Sorocaba, SP, Brazil 2; leonardo.fraceto@unesp.br

* Correspondence: vanessatakeshita@usp.br

Abstract: Nanoformulations have been used to improve the delivery of fertilizers, pesticides and growth regulators, with a focus on more sustainable agriculture. Nanoherbicide research has focused on efficiency gains through targeted delivery and environmental risk reduction. However, research on the behavior and safety of the application of these formulations in cropping systems is still limited. Organic matter contained in cropping systems can change the dynamics of herbicide-soil interactions in the presence of nanoformulations. The aim of this study was to use classical protocols from regulatory studies to understand the retention and mobility dynamics of a metribuzin nanoformulation, compared to a conventional formulation. We used different soil systems and soil with added fresh organic material. The batch method was used for sorption-desorption studies and soil thin layer chromatography for mobility studies, both by radiometric techniques. Sorption parameters for bothformulations showed that retention is a reversible process in all soil systems (H ~1.0). In deep soil with added fresh organic material, nanoformulation was more sorbed (14.61±1.41%) than commercial formulation (9.72±1.81%) ($p<0.05$). However, even with the presence of straw as a physical barrier, metribuzin in nano and conventional formulations was mobile in the soil, indicating that the straw can act as a barrier to reduce herbicide mobility but is not impeditive to herbicide availability in the soil. Our results suggest that environmental safety depends on organic material maintenance in the soil system. The availability can be essential for weed control, associated with nanoformulation efficiency, in relation to the conventional formulation.

Keywords: nanoformulation; nanoherbicide; sorption-desorption; soil mobility; soil organic matter

Table S1. Sorption-desorption parameters for *nano*MTZ and MTZ in different soil systems. Data indicates mean ± standard error. As interaction within soil systems and formulation was not significant, lowercase letters indicate differences between soil systems and ns indicates no significance within formulation, by Tukey's test (p<0.05).

		**Parameters**			
**Variables**		Sorption [a] (%)	K_{OC} sorption (mL g^{-1})	Desorption [b] (%)	K_{OC} desorption (mL g^{-1})
Formulation	*nano*MTZ	52.91 ± 4.68 ns	97.61 ± 12.55 ns	37.54 ± 3.89 ns	159.87 ± 16.3 ns
	MTZ	52.76 ± 4.92 ns	100.08 ± 12.18 ns	38.37 ± 4.32 ns	159.69 ± 30.37 ns
Soil systems	NC	63.27 ± 0.92 a	86.54 ± 3.4 b	29.87 ± 1.11 b	130.29 ± 14.46 b
	SC-CT	47.27 ± 1.2 c	98.24 ± 4.9 b	41.9 ± 2.11a	159.83 ± 15.87 ab
	SC-NT	51.70 ± 2.23 b	96.22 ± 8.14 b	39.38 ± 4.64 a	150.04 ± 25.35 ab
	SG-MN	49.13 ± 1.36 bc	120.41 ± 6.42 a	40.67 ± 3.38 a	198.98 ± 33.28 a

[a]Calculated in relation of total applied

[b]Calculated in relation of total sorbed

Table S2. Correlation matrices for *nano*MTZ and MTZ between sorption-desorption processes and soil characteristics of different soil systems. The values correspond to Pearson's Correlation Factor (r). Numbers with ^{ns} represents non-significant correlation and those with * are significant, at 5% of significance (p<0.05).

		Pearson's Correlation Coefficient (r)											
	Parameters	Sorption	Kd sorption	Desorption	Kd desorption	Sand	Silt	Clay	OC	CEC	SB	OM	pH
*nano*MTZ	Sorption	1	0.99*	-0.99*	0.98*	0.39 ^{ns}	0.99*	-0.92 ^{ns}	0.96*	0.88 ^{ns}	0.86 ^{ns}	0.96*	-0.16 ^{ns}
	Kd sorption		1	-0.99*	0.99*	0.45 ^{ns}	0.99*	-0.94 ^{ns}	0.97*	0.91 ^{ns}	0.90 ^{ns}	0.97*	-0.08 ^{ns}
	Desorption			1	-0.99*	-0.47 ^{ns}	-0.98*	0.95*	-0.98*	-0.92 ^{ns}	-0.90 ^{ns}	-0.98*	0.13 ^{ns}
	Kd desorption				1	0.51 ^{ns}	0.98*	-0.96*	0.98*	0.93 ^{ns}	0.93 ^{ns}	0.98*	-0.02 ^{ns}
	Sand					1	0.34 ^{ns}	-0.71 ^{ns}	0.61 ^{ns}	0.77 ^{ns}	0.77 ^{ns}	0.61 ^{ns}	0.38 ^{ns}
	Silt						1	-0.89 ^{ns}	0.94 ^{ns}	0.85 ^{ns}	0.84 ^{ns}	0.94 ^{ns}	-0.16 ^{ns}
	Clay							1	-0.99*	-0.99*	-0.98*	-0.99*	-0.05 ^{ns}
	OC								1	0.97*	0.96*	0.99*	-0.01 ^{ns}
	CEC									1	0.99*	0.97*	0.10 ^{ns}
	SB										1	0.96*	0.22 ^{ns}
	OM											1	-0.02 ^{ns}
	pH												
MTZ	Sorption	1	0.99*	-0.98*	0.98*	0.52 ^{ns}	0.97*	-0.96*	0.98*	0.94 ^{ns}	0.94 ^{ns}	0.98*	0.01 ^{ns}
	Kd sorption		1	-0.98*	0.98*	0.52 ^{ns}	0.97 *	-0.96*	0.98*	0.94 ^{ns}	0.9 ^{ns}	0.98*	0.02 ^{ns}
	Desorption			1	-0.99*	-0.48 ^{ns}	-0.95*	0.93 ^{ns}	-0.95*	-0.91 ^{ns}	-0.93 ^{ns}	-0.95*	-0.13 ^{ns}
	Kd desorption				1	0.42 ^{ns}	0.97 *	-0.91 ^{ns}	0.95*	0.88 ^{ns}	0.90 ^{ns}	0.95*	0.07 ^{ns}
	Sand					1	0.34 ^{ns}	-0.71 ^{ns}	0.61 ^{ns}	0.78 ^{ns}	0.77 ^{ns}	-0.61 ^{ns}	0.38 ^{ns}
	Silt						1	-0.89 ^{ns}	0.94 ^{ns}	0.85 ^{ns}	0.84 ^{ns}	0.94 ^{ns}	-0.16 ^{ns}
	Clay							1	-0.99*	-0.99*	-0.98	-0.99*	-0.05 ^{ns}
	OC								1	0.97*	0.96*	0.99*	-0.02 ^{ns}
	CEC									1	0.99*	0.97*	0.10 ^{ns}
	SB										1	0.96*	0.22 ^{ns}
	OM											1	-0.02 ^{ns}
	pH												1

Table S3. Sorption-desorption parameters for *nano*MTZ and MTZ in soil with addition of different organic residues. Data indicates mean ± standard error. As interaction within soil systems and formulation was not significant, lowercase letters indicate differences between organic residues (regarding formulation) and uppercase letters indicate differences between formulation (regarding organic residues), by Tukey's test ($p < 0.05$).

**Variables**		Sorption [a] (%)	Desorption [b] (%)
Formulation	*nano*MTZ	14.61 ± 1.41 A	71.07 ± 0.58 B
	MTZ	9.72 ± 1.81 B	79.46 ± 0.66 A
Organic residues	Soil - control	10.88 ± 3.28 bc	71.12 ± 1.05 c
	Black oat	12.6 ± 2.63 ab	73.54 ± 1.81 c
	Forage turnip	11.22 ± 3.28 c	74.67 ± 1.77 bc
	Sugarcane	13.37 ± 2.76 a	73.18 ± 1.63 c
	Corn	11.52 ± 3.19 bc	77.54 ± 1.59 ab
	Cassava	12.06 ± 2.58 abc	78.41 ± 1.3 a

[a]Calculated in relation of total applied

[b]Calculated in relation of total sorbed

Table S4. Retention factor (Rf) to *nano*MTZ and conventional MTZ soil mobility, obtained through soil thin layer chromatography (soil-TLC). Values represent the mean ± standard error.

		Retention fator (Rf)	
		Herbicide formulation	
		*nano*MTZ	MTZ
Organic residues	Soil - Control	0.88 ± 0.03	0.98 ± 0.03
	Black oat	0.80 ± 0.04	0.95 ± 0.03
	Forage turnip	0.88 ± 0.03	0.93 ± 0.03
	Sugarcane	0.90 ± 0.00	0.93 ± 0.03
	Corn	0.80 ± 0.00	0.85 ± 0.06
	Cassava	0.85 ± 0.09	0.90 ± 0.00
Soil systems	NC	0.45 ± 0.03	0.50 ± 0.00
	SC-NT	0.60 ± 0.00	0.53 ± 0.03
	SC-CT	0.58 ± 0.03	0.60 ± 0.00
	SG-MN	0.63 ± 0.03	0.63 ± 0.03
	DS	0.98 ± 0.03	1.0 ± 0.00

Table S5. Soil physicochemical properties from different crop and non-crop system. The soil samples correspond to non-crop soil (NC), cultivated soil with soybean-corn succession in a no-tillage system (SC-NT), cultivated soil with soybean-corn succession in a conventional tillage system (SC-CT), sugarcane monoculture soil (SG-MN), and deep soil (DS) to fresh organic materials addition.

**Parameters**[1]	**Soil**				
	**NC**	**SC-NT**	**SC-CT**	**SG-MN**	**DS**
Soil texture	Clay	Clay	Clay	Clay	Clay
Total sandy (g kg^{-1})	198	193	148	115	185
Silt (g kg^{-1})	303	159	213	193	134
Clay (g kg^{-1})	499	648	638	691	681
pH ($CaCl_2$)	5.7	5.9	5.2	5.7	4.3
OM (g dm^3)	68.7	31.5	38.5	27.7	5.9
OC (%)	4.0	1.9	2.2	1.7	0.3
P (mg dm^3)	46.1	67.2	14.7	20.4	<7
K (mmolc dm^3)	9.6	5.3	2.8	4.8	1.9
Ca (mmolc dm^3)	149.6	81.0	71.0	39.8	21.0
Mg (mmolc dm^3)	29.1	13.2	10.1	20.5	8.2
H+Al (mmolc dm^3)	24.7	21.3	34.3	15.8	43.1
SB[2] (mmolc dm^3)	188.3	99.5	83.9	65.1	31.1
CEC[3] (mmolc dm^3)	213	120.8	118.1	80.9	74.2
V[4] (%)	82	82	71	80	42

[1] Soils analyzed at the Laboratory of Mineral Fertilizers of the Superior School of Agriculture "Luiz de Queiroz", University of São Paulo, Piracicaba, São Paulo, Brazil.

[2] Sum of basis.

[3] Cation exchange capacity.

Table S6. Physical-chemical properties of organic residues added to soil.

Parameter	Black oat	Forage turnip	Sugarcane	Corn	Cassava
pH ($CaCl_2$)	6.10	5.70	6.3	7.4	4.7
C/N ratio	24	12	90	47	79
Density (g cm^{-3})	0.20	0.29	0.13	0.2	0.41
Organic matter (%)	73.28	72.54	77.86	90.01	96.17
Organic carbon (%)	40.71	40.30	42.10	48.45	52.11
Total Nitrogen (%)	1.68	3.24	0.47	1.03	0.66
Total phosporus (%)	0.42	1.28	0.21	0.36	0.11

Total potassium (%)	2.34	3.72	0.33	1.35	0.17
Total calcium (%)	1.03	3.08	0.98	0.37	0.3
Total magnesium (%)	0.22	0.67	0.13	0.25	0.06
Total sulfur (%)	0.11	0.53	0.26	0.31	0.15
